# Chemical Constituents Comparison Between the Flowers of *Sophora japonica* L. and *Robinia pseudoacacia* L. by UPLC-Q-TOF-MS/MS and HPLC

**DOI:** 10.3390/molecules31081238

**Published:** 2026-04-09

**Authors:** Cui-Cui Sun, Yi-Ting Chen, Hai-Xia Xu, Yu-Xian Guo, Qing-Feng Zhang

**Affiliations:** 1 School of Public Health and Health Management, Gannan Medical University, Ganzhou 341000, China; 2 Jiangxi Key Laboratory of Natural Product and Functional Food, College of Food Science and Engineering, Jiangxi Agricultural University, Nanchang 330045, Chinalewis_xust@126.com (Y.-X.G.)

**Keywords:** *Sophora japonica* L. flowers, *Robinia pseudoacacia* L. flowers, chemical constituents, flavonoids

## Abstract

The flowers of *Sophora japonica* L. (SJF) and *Robinia pseudoacacia* L. (RPF) are edible and similar in appearance. The chemical constituents of SJF and RPF were compared by UPLC-Q-TOF-MS/MS and HPLC analysis in this study. A total of 29 and 19 constituents were identified in SJF and RPF, respectively. Flavonoid glycosides were the main constituents found in both flowers. The main aglycon moieties found in SJF were quercetin, kaempferol and isorhamnetin, whereas acacetin and kaempferol were the main ones found in RPF. Additionally, the content of flavonoids in SJF was significantly higher than that in RPF, as determined by HPLC. Rutin was the most dominant flavonoid in SJF with a content range of 72.31~88.15 mg/g, followed by quercetin (13.05~20.30 mg/g). Kaempferol-di(rhamnoside)-hexoside was the most dominant flavonoid in RPF with a content range of 25.94~30.00 mg/g. The distinct flavonoid profiles indicated the chemical non-equivalence of SJF and RPF. Therefore, RPF should not be considered a direct substitute for SJF in herbal medicine without further pharmacological and clinical validation.

## 1. Introduction

**Figure 1:**
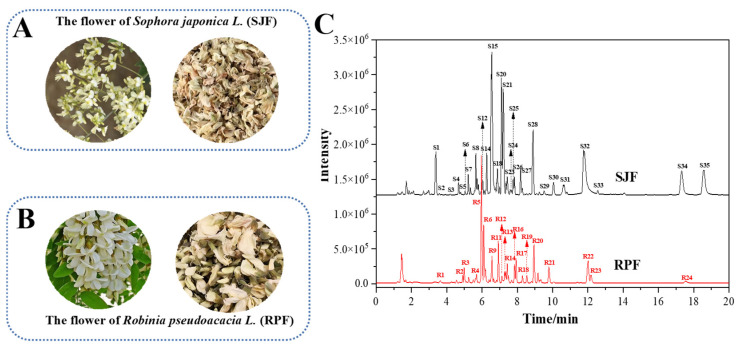
Morphological diagram of SJF (**A**) and RPF (**B**), and the BPCs of SJF and RPF analyzed by UPLC-Q-TOF-MS/MS (**C**).

*Sophora japonica* L. (*S*. *japonica*), belonging to the leguminous family, Sophora genera, is a tree originating in northern China. *Robinia pseudoacacia* L. (*R*. *pseudoacacia*), belonging to the leguminous family, Robinia genera, is a tree native to North America and introduced to China in the late 18th century. Normally, the fresh flower of *S*. *japonica* (SJF) and *R*. *pseudoacacia* (RPF) can be preliminarily distinguished by their flower color. SJF are mostly yellow-white or light yellow, while RPF are completely white (Figure 1A,B). However, after drying, these two materials become extremely similar in appearance, and both are even named “huaihua” in Chinese. Consequently, dried SJF and RPF are often confused and sold together in the market. Therefore, especially for powdered or processed materials, the reliability of macroscopic identification decreases, while chemical analysis provides an objective supplementary method.

The term “huaihua” in the Chinese Pharmacopoeia specifically refers to SJF, which is recognized for its superior medicinal properties. The main pharmacological actions of SJF recorded in the Chinese Pharmacopoeia are “clear heat and cool the blood” with treatments for hemorrhoids, hemafecia, hematemesis, etc. [1]. Modern pharmacological studies have demonstrated that SJF contain various flavonoids, such as rutin, quercetin, nicotiflorin, kaempferol, and isorhamnetin [2,3]. The Chinese Pharmacopoeia requires the rutin content to be greater than 6% in dried SJF and greater than 15% in the flower buds [1]. The flavonoid-rich SJF extracts exhibit bioactivities of anti-diabetes [4], anti-hyperuricemia [5], anti-inflammation [6], etc. In contrast to the medicinal use of SJF, RPF are commonly eaten as fresh flowers with sweet fragrant odors. Currently, studies on the phytochemicals and bioactivities of RPF have been insufficient. Bhalla et al. investigated the chemical composition and antibacterial activity of RPF essential oil [7]. The nutritional attributes and total phenolic content of RPF were analyzed by Tian et al. [8]. Furthermore, RPF extract significantly ameliorated hypoxia-induced gastric and small intestinal mucosal injury in mice [9]. Uzelac et al. revealed that RPF extracts contained a high proportion of flavones and exhibited antimicrobial and cytotoxic activities [10].

Compared with the numerous studies on SJF, the exploration of the chemical components in RPF remains limited. Previous studies on SJF and RPF have mainly focused on the total phenolic and flavonoid contents, as well as the biological activities of their extracts. However, there is a lack of systematic comparative studies on the flavonoid profiles of the two flowers, particularly regarding the types and contents of individual monomeric flavonoids. Although the two flowers are similar in appearance, it is still unclear whether RPF can replace SJF in the field of medicine. Therefore, in this study, UPLC-Q-TOF-MS/MS was used to compare the chemical composition characteristics of SJF and RPF. Furthermore, HPLC was also employed to determine the content of the main flavonoid components.

## 2. Results and Discussion

### 2.1. Constituents Identification Through UPLC-Q-TOF-MS/MS Analysis

**Figure 2:**
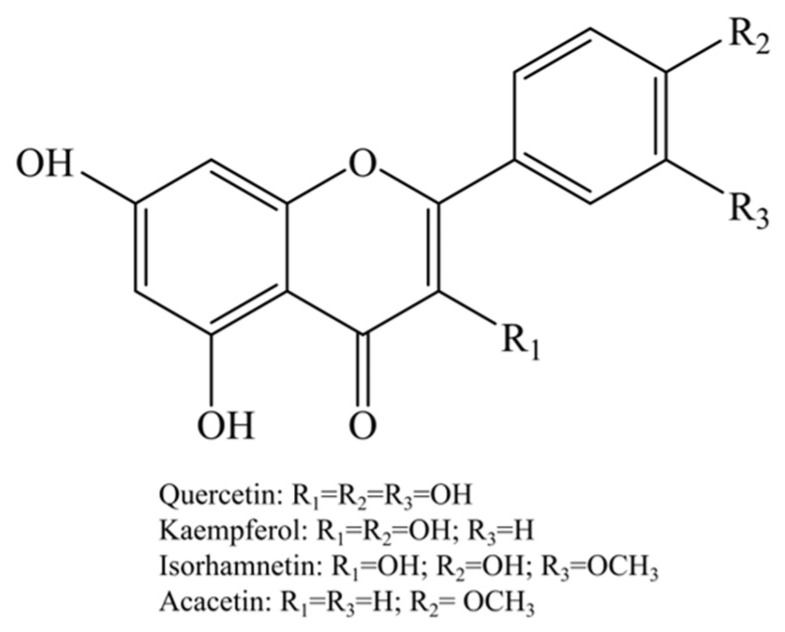
The main aglycon moiety of flavonoid found in SJF and RPF.

UPLC-Q-TOF-MS/MS analysis was conducted to identify the constituents in SJF and RPF. Figure 1 shows the morphological diagram of the two flowers and their base peak chromatograms (BPCs) analyzed by UPLC-Q-TOF-MS/MS. As shown, their chemical profiles were distinctly different. With the assistance of the Q-TOF MS detector, the information of a deprotonated molecule ([M−H]^−^) and its fragment ions of each peak were obtained. In combination with a comparison of the literature, their chemical structures were preliminarily deduced. Table 1 and Table 2 present the comprehensive information of each peak. A total of 29 constituents were successfully identified in SJF (Table 1), while 19 constituents were identified in RPF (Table 2). Among them, flavonoids were the main constituents found in both flowers. Flavonoids in plants are commonly presented as glycosides and typically generate the base peak ions of their aglycons through natural loss of their sugar moiety in MS analysis. The main sugar moiety found in flavonoid glycosides includes rutinose (−308 D), hexose (such as glucose (−162 D), galactose (−162 D), rhamnose (−146 D)) and pentose (−132 D). Furthermore, based on the fragmentation rules of flavonoid aglycons, their structures could be deduced [11,12]. Most importantly, flavonoid aglycons with 5-OH and 7-OH in the A ring could produce a fragment ion of *m*/*z* 151 through the retro-Diels-Alder fragmentation mechanism [11]. For example, rutin showed a deprotonated molecule [M−H]^−^ at *m*/*z* 609. The based ion peak at *m*/*z* 300 was its aglycon, quercetin, which was produced due to the neutral loss of rhamnose moiety. Quercetin can further produce characteristic ions such as *m*/*z* 271, 255, 151, etc. Based on the fragmentation rules and literature validation, the chemical structures of the four main flavonoid aglycons found in SJF and RPF, i.e., quercetin, kaempferol, isorhamnetin and acacetin, are shown in Figure 2. Their deprotonated molecules [M−H]^−^ were found at *m*/*z* 301/300, 285, 315, and 283, respectively. In the study by Xie et al., the constituents in SJF were identified by HPLC-DAD-ESI-MS/MS [2]. It was also found that quercetin and its derivatives accounted for most constituents in SJF, followed by isorhamnetin and kaempferol derivatives.

**Figure 3:**
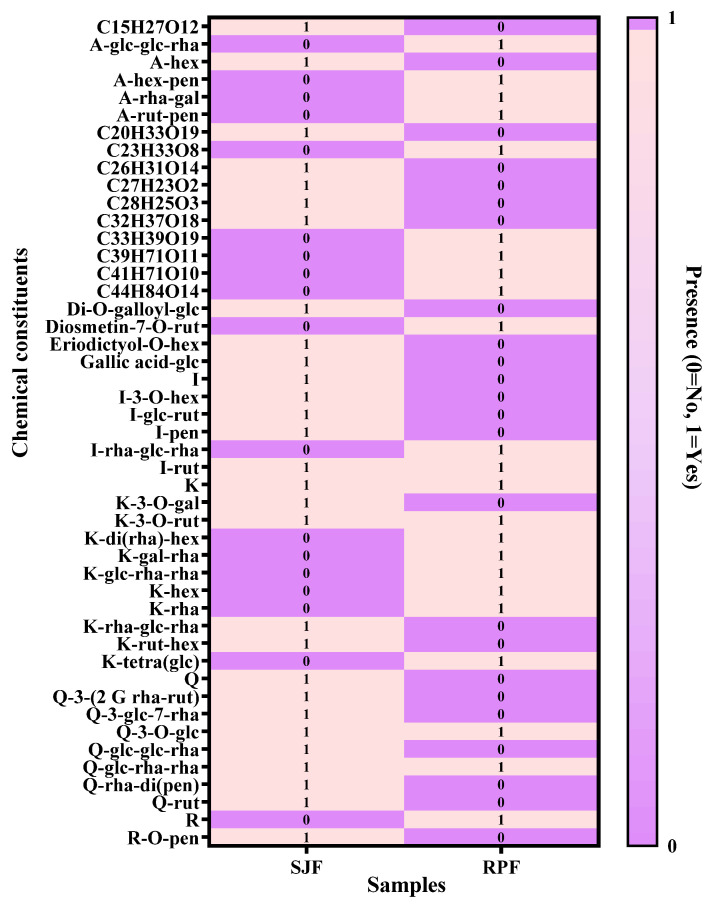
Heatmap of the chemical components in SJF and RPF.

A comparative heatmap analysis was conducted based on the identification results obtained by UPLC-Q-TOF-MS/MS. As shown in Figure 3, there were significant differences in the chemical compositions of these two plant species. Among all the identified components, only five compounds were shared by both (Isorhamnetin-rutinoside, kaempferol, quercetin-glucoside-rhamnoside-rhamnoside, quercetin-3-O-glucoside, and kaempferol-3-O-rutinoside). The Q-TOF and heatmap analysis results revealed that flavonoid glycosides were the main phytochemicals in both SJF and RPF. However, the main aglycons in SJF were quercetin, kaempferol, and isorhamnetin, while those in RPF were acacetin and kaempferol.

### 2.2. Quantification of Main Flavonoids in SJF and RPF by HPLC

**Figure 4:**
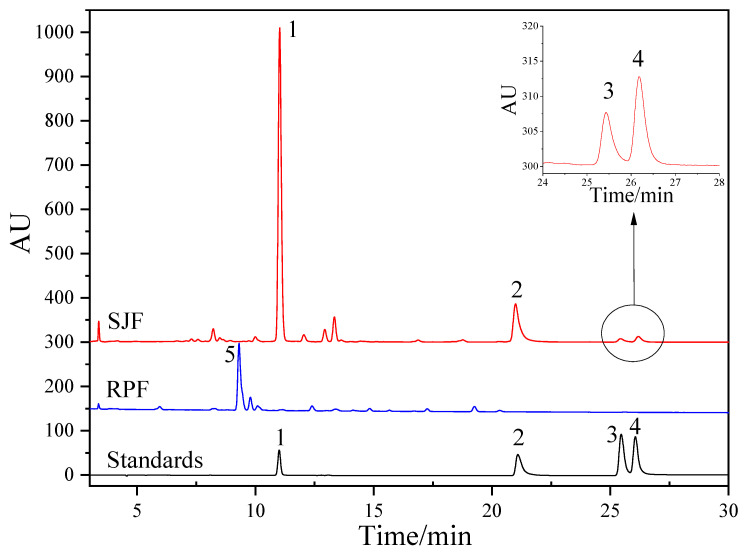
The HPLC chromatogram of SJF, RPF and standard markers. Peaks: 1, rutin; 2, quercetin; 3, kaempferol; 4, isorhamnetin; 5, kaempferol-di(rhamnoside)-hexoside.

The HPLC chromatograms of SJF and RPF are presented in Figure 4. Similarly to the UPLC-Q-TOF-MS analysis, the chemical profiles of the two species were completely different. Almost no peak with the same retention time was found between them. Meanwhile, the peak abundance in RPF was significantly lower than that in SJF, indicating the lower flavonoid content of RPF. Four commercially available flavonoid markers, namely rutin, quercetin, kaempferol, and isorhamnetin, were separated by HPLC under the same conditions. By comparing with the retention time and UV spectra, the peaks of these four flavonoids in the chromatogram of SJF were successfully identified. Rutin, with the biggest peak area, was the most dominant flavonoid in SJF, followed by quercetin. UPLC-Q-TOF-MS analysis showed that rutin and kaempferol were present in RPF. However, no distinct peaks of these constituents were found in the HPLC chromatogram of RPF. The results reflect the significantly low levels of these constituents in RPF and the relatively limited sensitivity of the HPLC UV detector. The HPLC quantitative method for the determination of rutin, quercetin, kaempferol, and isorhamnetin in SJF was developed. The method validation results are listed in Table 3. The contents of the four flavonoids in three batches of SJF were determined. The content range of rutin, quercetin, kaempferol and isorhamnetin was 72.31~88.15 mg/g, 13.05~20.30 mg/g, 0.42~0.90 mg/g and 0.55~1.16 mg/g, respectively. The Chinese Pharmacopoeia requires the content of rutin in SJF to be >60 mg/g [1]. Hence, all the tested SJF samples fulfilled the requirement.

According to the UPLC-Q-TOF-MS analysis results, the biggest peak observed in the HPLC chromatogram of RPF (Peak 5 in Figure 4) was identified as kaempferol-di(rhamnoside)-hexoside (Peak R5 in Figure 1C). It also exhibited a UV spectrum similar to that of the kaempferol marker. However, this particular flavonoid was commercially unavailable. Hence, its content in RPF was determined by using the calibration curve of kaempferol with the consideration of its molecular weight. The content range of kaempferol-di(rhamnoside)-hexoside in the three batches of RPF was 25.94~30.00 mg/g. Quantitative analysis revealed that the flavonoid content in RPF was significantly lower than that in SJF. The total flavonoids content in SJF was about 3~4 times that in RPF.

Flavonoids exhibit various health-promoting bioactivities [30]. A recent epidemiologic study revealed that diverse consumption of flavonoids significantly lowered (6~20%) the risk of all-cause mortality and incidence of various chronic diseases [31]. Hence, when consumed as dietary material, both RPF and SJF could provide substantial health-promoting effects. Although the two are similar in appearance and both contain flavonoids, they have completely different chemical composition spectra in terms of the types and contents of flavonoids. This chemical divergence directly translates into significant pharmacological and clinical differences, which strictly limit their interchangeable use in traditional herbal medicine. The traditional medicinal value of SJF is primarily attributed to its ability to “clear heat and cool blood,” and it is commonly used to treat hemorrhagic and inflammatory diseases such as hemorrhoids and hematochezia [1]. This therapeutic effect is highly dependent on its flavonoid component profile, primarily composed of rutin and quercetin. The modern pharmacological literature has confirmed that rutin and quercetin have specific abilities to enhance the resistance of capillaries and exert systemic hemostatic effects, as well as powerful anti-inflammatory, antioxidant, anti-diabetes, and anti-hyperuricemia effects [32,33]. This not only directly confirms the scientific connotation of the traditional uses of SJF in the Chinese Pharmacopoeia but also complies with the Pharmacopoeia’s mandatory quality standard that the content of rutin in the dried product must be >60 mg/g (6%). In contrast, the UPLC-Q-TOF-MS/MS and HPLC analysis results of this study showed that the types of flavonoids in RPF were fewer, and the total flavonoid content was 3 to 4 times lower than that of SJF. More importantly, rutin and kaempferol monomers could not be detected in RPF under conventional HPLC testing. Moreover, the overall component spectrum of RPF was dominated by acacetin and kaempferol derivatives. Recent pharmacological reviews have indicated that acacetin is a highly promising multi-target active substance, exhibiting properties such as lipid-regulating, anticancer, anti-inflammatory, neuroprotective, and cardiovascular protective effects [34]. However, its pharmacological mechanism differs from that of rutin, which mainly targets vascular protection and hemostasis.

From the perspective of clinical medication, confusion regarding the drug varieties will result in patients being unable to obtain the highly active targeted compounds to required achieve therapeutic dosages. Although RPF has high potential for the development of nutritious and functional foods, it cannot be regarded as an equivalent substitute for SJF in traditional pharmacy from the perspective of pharmacological equivalence. Therefore, the results of this study can provide a research basis for the quality control of the “huaihua” herb and help to establish a fingerprint pattern or content standard based on multiple flavonoid components (e.g., rutin, quercetin, kaempferol, isorhamnetin), ensuring the authenticity and consistency of the herb’s efficacy.

## 3. Materials and Methods

### 3.1. Materials and Reagents

Six batches of dried SJF and RPF samples, with three for each, were bought from Nanchang drugstore or online shops. The plant materials were authenticated by Prof. Zhong-Ping Yin (Jiangxi Agricultural University), and the voucher specimens were deposited in Jiangxi Key Laboratory of Natural Product and Functional Food with number of JXNPF-216 to JXNPF-221. Reference specimen of SJF was bought from National Institutes for Food and Drug Control (Beijing, China).

HPLC-grade acetic acid and acetonitrile were obtained from Anhui Tedia High Purity Solvent Co., Ltd. (Anqin, China). Rutin, quercetin, kaempferol and isorhamnetin (purity > 98%) were purchased from Aladdin Biochemical Technology Co., Ltd. (Shanghai, China). All other reagents used in this research were of analytical grade.

### 3.2. Sample Preparation and Extraction

SJF and RPF were pulverized into powder and sieved through a 60-mesh sieve. An aliquot of 0.1 g sample was mixed with 25 mL of 60% ethanol. After sonication for 30 min, the extract was centrifuged at 5000 r for 10 min. The supernatant was filtered through a 0.22 μm filter membrane before analysis.

### 3.3. UPLC-Q-TOF-MS/MS Analysis

The UPLC-Q-TOF-MS/MS analysis was conducted using a Q-TOF 5600-plus mass spectrometer equipped with Turbo V sources and a Turbolonspray interface (AB Sciex Corporation, Foster City, CA, USA), coupled with a Shimadzu LC-30A UPLC system (Shimadzu Corporation, Kyoto, Japan). An acquity UPLC BEH C18 column (2.1 mm × 100 mm, 1.7 μm, Waters, Milford, MA, USA) was used for separation. The flow rate was set at 0.3 mL/min while the injection volume was 3 μL; the column temperature was kept constant at 40 °C. The mobile phase employed was the same as HPLC analysis. The mass spectrometer was operated in negative ion mode, with ultrapure nitrogen serving as ion source gas 1 (50 psi), ion source gas 2 (50 psi), and curtain gas (40 psi). Turbo Ion Spray voltage and temperature were set at −4500 V and 500 °C, respectively. Declustering potential, collision energy, and collision energy spread were established at 100 V, −40 V, and 10 V, correspondingly. Data acquisition was executed using Analyst 1.6 software from AB Sciex.

### 3.4. HPLC Analysis

Agilent 1260 HPLC system and Agilent 5 TC-C18 column (250 × 4.6 mm, 5 μm, Santa Clara, CA, USA) were employed. Mobile phases consisted of acetonitrile (A) and 0.2% acetic acid solution (B) with a flow rate of 1 mL/min and linear gradient program of 15.0–50.0% (A) in 0–30 min. The column temperature was set at 40 °C, and injection volume was 10 μL. Detection wavelength was set at 355 nm.

### 3.5. Statistical Analysis

All quantitative experiments had at least three replicates, and the data were expressed as mean ± standard deviation (SD).

## 4. Conclusions

UPLC-Q-TOF-MS/MS analysis showed that flavonoid glycosides were the main constituents found in both SJF and RPF. However, the main aglycon moieties found in SJF were quercetin, kaempferol, and isorhamnetin, while those in RPF were acacetin and kaempferol. Furthermore, HPLC quantitative analysis revealed that the flavonoid content in SJF was significantly higher than that in RPF. The flavonoid profile of SJF also exhibited greater diversity than that of RPF. In summary, SJF and RPF showed distinct flavonoid profiles in both composition and content. Therefore, RPF should not be regarded as chemically equivalent to SJF. And chromatographic analysis is helpful in distinguishing these two materials in commercial circulation. However, further comparative bioactivity studies are needed before drawing conclusions regarding therapeutic substitution.

## Figures and Tables

**Table 1:** Chemical constituents in SJF analyzed by UPLC-Q-TOF-MS/MS.

Peak	t_R_ (min)	[M−H]^−^	Major and Important MS^2^ Ions	Identification	Reference
S1	3.374	399.1513	207(100), 353(34), 161(26), 101(24)	C_15_H_27_O_12_ *	
S2	3.561	331.0619	169(100), 125(60)	Gallic acid-glc	[13]
S3	4.203	447.1160	152(72), 108(10), 315(4), 271(3)	I-pen	
S4	4.673	483.0787	169(100), 331(61), 125(51), 287(30), 439(23), 313(21), 271(7)	Di-O-galloyl-glc ^a^	[14]
S5	4.804	771.2022	771(100), 609(50), 463(16), 301(12), 271(2)	Q-glc-glc-rha ^b^	[15]
S6	5.033	483.0788	169(100), 331(36), 125(17), 313(5), 151(2)	Di-O-galloyl-glc ^a^	[14]
S7	5.218	563.1631	419(100), 461(31), 125(29), 161(24), 501(10), 101(10), 203(8)	C_26_H_31_O_14_ *	
S8	5.647	771.2016	300(36), 178(5), 271(5), 591(4), 255(2), 609(3)	Q-glc-glc-rha ^b^	[15]
S9	5.726	755.2026	609(56), 446(17), 301(14)	Q-3-(2 G rha-rut) ^c^	[16]
S10	5.805	609.1474	446(100), 299(62), 463(29), 271(8)	Q-3-glc-7-rha ^d^	[15]
S11	5.949	755.2066	755(100), 300(32), 271(3)	Q-glc-rha-rha ^c^	[15]
S12	6.035	785.2175	785(100), 314(20), 300(6), 623(2), 605(4), 271(2)	I-glc-rut	
S13	6.124	739.2117	593(100), 739(36), 285(27), 431(10)	K-rha-glc-rha	
S14	6.269	741.1912	301(28), 271(3), 609(2), 179(2)	R-O-pen	[17]
S15	6.543	609.1473	301(100), 609(79), 271(14), 255(8), 179(7), 151(6)	Q-rut ^d^	[18]
S16	6.743	447.1049	285(100), 447(22)	K-3-O-gal ^e^	[19]
S17	6.822	755.2066	755(100), 315(50), 300(5), 271(2), 623(1)	K-rut-hex	
S18	6.854	463.0889	300(100), 271(22), 255(13)	Q-3-O-glc	[20]
S19	6.996	610.4197	564(100), 225(6), 300(2)	Q-rha-di(pen)	
S20	7.092	593.1518	285(100), 255(16), 227(8), 151(2)	K-3-O-rut	[15]
S21	7.156	623.1632	315(100), 300(20), 271(10), 243(5)	I-rut	[21]
S22	7.360	447.0942	285(100)	K-3-O-glc ^e^	[19]
S23	7.424	609.1480	301(100), 151(11), 178(10)	Q-rut ^d^	[18]
S24	7.598	477.1048	314(100), 271(32), 243(28), 300(12)	I-3-O-hex	[22]
S25	7.758	609.1482	301(100), 609(22), 178(8), 151(8)	Q-rut ^d^	[18]
S26	7.853	449.1095	151(100), 287(72), 135(30), 107(4), 125(3)	Eriodictyol-O-hex	[17]
S27	8.187	709.2007	145(100), 565(58), 419(46), 607(43), 163(36), 461(20)	C_32_H_37_O_18_ *	
S28	8.885	577.1582	145(100), 163(78), 475(37), 433(36), 307(26), 515(23)	C_20_H_33_O_19_ *	
S29	9.501	379.0674	119(100), 259(60), 216(17), 233(7)	C_27_H_23_O_2_ *	
S30	10.048	409.1778	119(100), 134(95), 259(67), 149(66), 289(51), 393(6)	C_28_H_25_O_3_ *	
S31	10.500	609.1476	300(76), 271(10), 255(5), 151(4)	Q-rut ^d^	
S32	11.792	301.0367	151(100), 121(43), 178(33), 271(12)	Q	[23]
S33	12.533	445.1142	283(100), 268(34), 151(4)	A-hex	
S34	17.319	285.0417	285(100), 185(18), 239(14), 211(14), 159(12)	K	[24]
S35	18.569	315.0521	300(100), 271(30), 164(21), 255(20)	I	[25]

* Constituents with formula but not identified. Compounds abbreviations: A: acacetin; Q: quercetin; R: rutin; K: kaempferol; I: isorhamnetin. Glycoside abbreviations: glc: glucoside; gal: galactoside; rha: rhamnoside; rut: rutinoside; hex: hexoside: pen: pentoside. ‘a’, ‘b’, ‘c’, ‘d’, ‘e’ indicate that the compounds are isomers of each other.

**Table 2:** Chemical constituents in RPF analyzed by UPLC-Q-TOF-MS/MS.

Peak	t_R_ (min)	[M−H]^−^	Major and Important MS^2^ Ions	Identification	Reference
R1	3.632	437.2131	218(100), 146(22)	C_23_H_33_O_8_ *	
R2	4.910	755.2038	593(100), 285(8), 446(3)	K-glc-rha-rha	[15]
R3	4.986	901.2619	755(100), 285(10), 430(4), 575(2)	K-tetra(glc) ^f^	[26]
R4	5.693	755.2041	609(49), 446(17), 301(15), 271(2)	Q-glc-rha-rha	[15]
R5	5.966	739.2093	593(100), 285(22), 430(17), 327(4), 255(3)	K-di(rha)-hex	[27]
R6	6.015	593.1507	447(100), 430(85), 283(50), 255(8)	K-gal-rha	[28]
R7	6.102	739.2093	593(100), 285(34), 431(22)	C_33_H_39_O_19_ *	
R8	6.210	769.2200	623(100), 315(22), 461(9), 300(1)	I-rha-glc-rha	
R9	6.561	609.1457	609(100), 300(100), 271(14), 255(8)	R	[18]
R10	6.793	463.0872	300(100), 271(24), 255(12), 178(5)	Q-3-O-glc	[20]
R11	6.926	593.1509	284(100), 255(20), 227(7), 151(4)	K-3-O-rut	[15]
R12	7.158	623.1595	314(65), 299(20), 271(5), 151(2)	I-rut	[21]
R13	7.286	447.0929	284(100), 255(52), 227(35)	K-hex	[19]
R14	7.428	723.5026	677(100), 225(5), 659(3), 338(2)	C_39_H_71_O_11_ *	
R15	7.682	607.1662	299(100), 284(17)	Diosmetin-7-O-rut	[29]
R16	7.859	836.5869	790(100), 225(3)	C_41_H_71_O_10_ *	
R17	7.928	767.2043	283(100), 767(28), 268(12), 483(11)	A-hex-pen	
R18	8.292	949.6712	901(100), 949(40)	K-tetra(glc) ^f^	
R19	8.541	799.2310	283(100), 753(31), 268(6)	A-glc-glc-rha	
R20	8.943	577.1558	145(100), 163(72), 475(42), 433(39), 515(27), 307(22)	C_44_H_84_O_14_ *	
R21	9.793	769.2197	283(100), 723(16), 268(8), 591(2)	A-rut-pen	
R22	12.003	637.1764	283(100), 268(22), 591(3)	A-rha-gal	[28]
R23	12.181	431.0978	285(100), 151(49), 431(40), 257(39), 213(6), 229(5)	K-rha	[28]
R24	17.495	285.0407	185(10), 229(8), 151(6)	K	[24]

* Constituents with formula but not identified. Compounds abbreviations: A: acacetin; I: isorhamnetin; K: kaempferol; Q: quercetin; R: rutin. Glycoside abbreviations: glc: glucoside; gal: galactoside; rha: rhamnoside; rut: rutinoside; hex: hexoside: pen: pentoside. ‘f’ indicates that the compounds are isomers of each other.

**Table 3:** Precision, linearity and recovery of HPLC method of four flavonoid markers.

Standard	RSD/%		Regression Equation	Linear Range/(μg/mL)	Linearity (R)	Recovery Rate/%
	Retention Time	Peak Area				
Rutin	0.40	0.51	Y = 21.520X	1~100	0.9995	102.73 ± 0.62
Quercetin	0.12	1.14	Y = 27.586X	1~100	0.9995	95.42 ± 1.38
Kaempferol	0.24	0.87	Y = 45.400X	1~100	0.9999	104.64 ± 0.26
Isorhamnetin	0.11	0.79	Y = 50.245X	1~100	0.9999	96.32 ± 0.62

## Data Availability

The original contributions presented in this study are included in the article. Further inquiries can be directed to the corresponding author.
